# High species richness of sheep‐grazed sand pastures is driven by disturbance‐tolerant and weedy short‐lived species

**DOI:** 10.1002/ece3.70282

**Published:** 2024-09-08

**Authors:** Gergely Kovacsics‐Vári, Judit Sonkoly, Katalin Tóth, Andrea McIntosh‐Buday, Patricia Elizabeth Díaz Cando, Viktória Törő‐Szijgyártó, Nóra Balogh, Luis Roberto Guallichico Suntaxi, Francis David Espinoza Ami, Gábor Matus, Béla Tóthmérész, Péter Török

**Affiliations:** ^1^ Department of Ecology University of Debrecen Debrecen Hungary; ^2^ HUN‐REN‐UD Functional and Restoration Ecology Research Group Debrecen Hungary; ^3^ Department of Botany University of Debrecen Debrecen Hungary; ^4^ HUN‐REN‐UD Biodiversity and Ecosystem Services Research Group Debrecen Hungary; ^5^ Polish Academy of Sciences Botanical Garden‐Centre for Biological Diversity Conservation in Powsin Warszawa Poland

**Keywords:** disturbance, European steppes, functional groups, humped‐back curve, plant biomass, sheep grazing

## Abstract

We selected 15 sheep‐grazed sand pastures along a gradient of increasing grazing intensity to study the fine‐scale patterns of main biomass fractions (green biomass, litter) and that of plant species and functional groups (life forms and social behaviour types). We classified them into five grazing intensity levels based on stocking density, proximity to drinking and resting places and the number of faeces. We aimed to answer the following questions: (i) How does increasing intensity of sheep grazing affect the amount of green biomass, the species richness and their relationship in sand pastures? (ii) How does increasing intensity of sheep grazing affect the biomass of perennial and short‐lived graminoids and forbs? (iii) How does the disturbance value—expressed in the biomass ratio of disturbance‐tolerant and ruderal species—change along the gradient of grazing intensity? A unimodal relationship between green biomass and species richness was detected; however, the ordination (canonical correspondence analysis, CCA) showed no clustering of pastures subjected to the same levels of grazing intensity. Along the grazing intensity gradient we found an increasing trend in species richness and significant differences in green biomass (decreasing trend), litter (decreasing trend), graminoids (decreasing trend) and short‐lived forbs (increasing trend). We found an increasing amount of disturbance‐tolerant and ruderal species with increasing grazing intensity. We suggest that we might need to use multiple scales for sampling and a fine‐scale assessment of grazing intensity. Our findings might be instructive for pastures in densely populated regions, which are prone to the encroachment of disturbance‐tolerant and ruderal species.

## Graphical abstract

We analysed the fine scale biomass patterns of sheep‐grazed sand pastures along increasing grazing intensity. Along the grazing intensity gradient, we found an increasing trend for species richness and the biomass of short‐lived forbs; while green biomass, litter and graminoid biomass showed a decreasing trend. We found an increasing amount of disturbance tolerant and ruderal species with increasing grazing intensity.

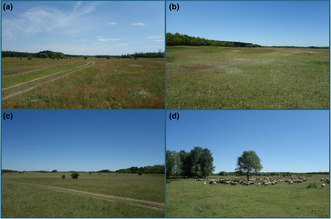

## 1 INTRODUCTION

It is a truism in ecology that grazing has a crucial role in maintaining grassland biodiversity (Briske, 1996; Metera et al., 2010), although it is a disturbance that affects both the morphological characteristics and the functional trait composition of plant communities (Díaz et al., 2007; WallisDeVries et al., 2002). To avoid overgrazing, it is essential to study the effects of grazing on species composition, vertical and horizontal structure, regeneration capacity and functional composition of plant communities (Dong et al., 2012; Hao & He, 2019). Overgrazing is considered to be one of the most pressing problems for implementing sustainable management of pastures in many regions of the world (Gao & Li, 2016; Li, Zhang, et al., 2018; Török & Dengler, 2018). Former research found that plant community responses along a gradient of grazing intensity showed marked changes, which led to altered stability and ecosystem functioning (Li et al., 2018b; Su & Xu, 2021; Xu et al., 2022). When studying the effects of grazing, grazing intensity, livestock type and habitat type can be regarded as the main approaches, and various combinations of these approaches can be found in the scientific literature.

Sheep grazing has some specific characteristics; for example, (i) there is a higher preference for forbs compared to cattle grazing, (ii) sheep can consume plant parts closer to the ground and (iii) sheep prefer vegetative plant parts (Jerrentrup et al., 2015; Metera et al., 2010; Tóth et al., 2018). Sheep grazing supports seedling establishment on bare soil surfaces in different ways: (i) Seeds lying on the soil surface can be buried to an optimal depth for germination by sheep trampling (Eichberg et al., 2005). (ii) Flocks usually consist of up to a few hundred sheep and are frequently herded by shepherds over relatively long distances; consequently, they can contribute to a wide dispersal of certain seeds (Rosenthal et al., 2012). (iii) Sheep trampling opens dense vegetation cover and creates safe sites for seedling emergence and establishment (Faust et al., 2011; Freund et al., 2014).

Species richness often shows a humped‐back curve along a gradient of increasing disturbance, which can be explained by the intermediate disturbance hypothesis (Connell, 1978; Gao & Carmel, 2020). This finding was also supported by studies dealing with sheep grazing (del Pozo et al., 2006; Lázaro et al., 2016; Süss et al., 2007). However, the spatial (Süss et al., 2007) and temporal (del Pozo et al., 2006) scale of the study can influence the relationship, and monotonously decreasing species richness has also been detected, e.g. in sheep‐grazed desert steppes (Zhang et al., 2018).

Besides patterns of biodiversity, a further important aspect is how changes in species richness are reflected in the abundance of plant life and growth forms. Díaz et al. (2007) analysed plant trait responses to grazing in a meta‐analysis. They found that increasing grazing intensity favoured stoloniferous plants, rosette‐formation likeliness and short height and increased the abundance of short‐lived and fast‐growing species when climatic conditions and grazing history were both taken into consideration. Some findings of the above‐mentioned meta‐analysis were partly confirmed for sheep‐grazed pastures by Pettit et al. (1995), Yang et al. (2022) and Farmilo et al. (2023). However, none of the above papers studied the biomass of life and growth form groups at multiple levels of grazing intensity in sheep‐grazed pastures. Compared to classical analyses of species composition and richness, functional trait‐ and functional group‐based analyses provide a more direct view for understanding the link between community assembly and functioning and how it is affected by livestock grazing (Carboni et al., 2023; Carmona et al., 2015).

Avoiding the encroachment of weedy and/or unwanted species in grazed grasslands is among the most important challenges of sustainable management (Bretas et al., 2023). While the formation of vegetation gaps by grazing is essential for the establishment of short‐lived pioneer species, it also provides colonisation windows for weeds and invasive species. To evaluate the effect of grazing intensity on the encroachment of weedy species is especially crucial for sand pastures as these types of grasslands are highly exposed to invasion (Botta‐Dukát, 2008). In some ecosystems, sheep grazing is an established method for supressing weedy forbs, mostly because sheep are considered to be highly selective grazers, which prefer forbs over grasses and shrubs (Jerrentrup et al., 2015; Olson & Lacey, 1994). However, it has also been stressed that the effect of livestock grazing is strongly context‐dependent and varying in different habitat types (Liu et al., 2015; Mládek et al., 2013; Török et al., 2024). Many sand grasslands in Central Europe are classified in the EU Habitats Directive as Pannonian and Pontic sandy steppes (E1.1a). These grasslands are located in Central and Southeast Europe and critically endangered according to the European red list of habitats (EC Directorate‐General for Environment et al., 2017), which underlines their conservation importance. For their sustainable management, it is crucial to understand how their vegetation composition and functional diversity respond to different grazing regimes.

With the study of sand pastures subjected to increasing intensity of sheep grazing, we aimed to address the following questions: (i) How does increasing intensity of sheep grazing affect the amount of green biomass, the species richness and their relationship in sand pastures? (ii) How does increasing intensity of sheep grazing affect the biomass of perennial and short‐lived graminoids and forbs? (iii) How does the disturbance value (expressed in the biomass ratio of disturbance‐tolerant and ruderal species) change along the gradient of grazing intensity?

## 2 MATERIALS AND METHODS

### 2.1 Study area

**FIGURE 1:**
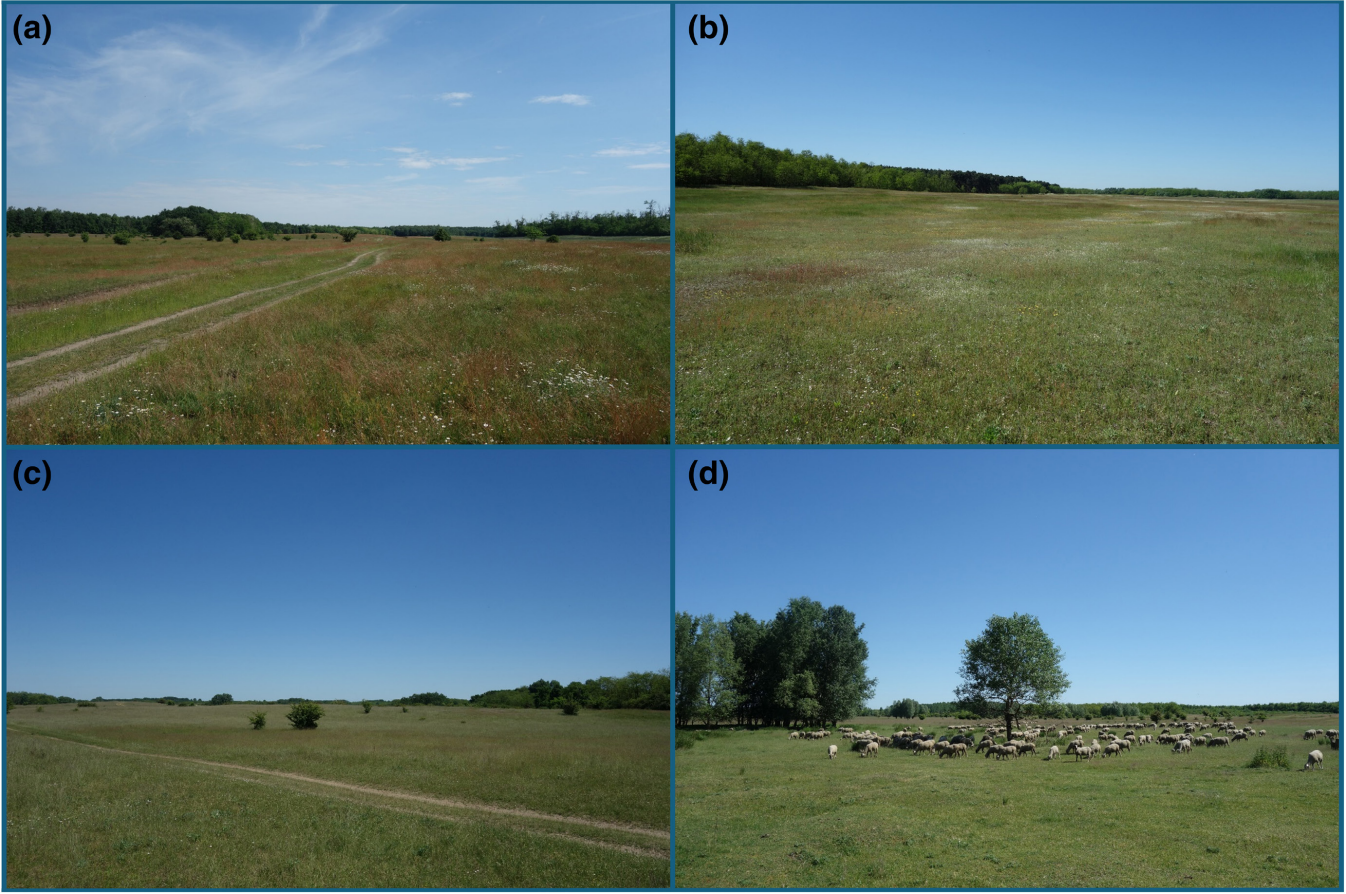
Typical view of sampled sand pastures in the surroundings of Hajdúbagos (a), Létavértes (b) and Hajdúsámson (Martinka district) (c, d), East Hungary. Photo credit: Judit Sonkoly.

For our study, we selected 15 sand pastures (Table 1) in the Nyírség region, East Hungary, where there is a high proportion of man‐made habitats such as croplands and tree plantations (Botta‐Dukát, 2008). The Nyírség region is characterised by an annual rainfall ranging between 530 and 680 mm and an average annual temperature between 9.4 and 9.8°C (Dövényi, 2010). In some years, the annual rainfall is even less than 400 mm so that serious drought events occur (Négyesi, 2018). The soil types are Dystric to Brunic Arenosols, which are characterised by a rather low humus content (0.6%–2.6% by mass) and a pH ranging typically from 4.45 to 5.71. Site codes are given in Table 1, further soil characteristics in Appendix S1. All investigated pastures are leased by the shepherds from the Hortobágy National Park Directorate except pasture 13, which is leased from the village authorities. The vegetation of the sand pastures is characterised by a high cover of tussock‐forming (*Festuca pseudovina*, *F. vaginata* and *Corynephorus canescens*) and stoloniferous (*Poa angustifolia*, *Cynodon dactylon*, *Carex praecox* and *C. stenophylla*) graminoids and a relatively high cover of short‐lived and perennial sand grassland forbs (*Scleranthus annuus*, *Vicia lathyroides*, *Potentilla arenaria*, *Rumex acetosella*, *Eryngium campestre* and *Chondrilla juncea*) (Figure 1).

**TABLE 1:** Main characteristics of the studied sites. The site codes were used in the canonical correspondence analysis (CCA) (Figure 5). The levels of grazing intensity (increasing from ‘1’ to ‘5’) are explained in Table 2.

Site code	Settlement name	GPS coordinates		Elevation (m)	Area of pasture (ha)	Level of grazing intensity
1	Létavértes	N47.41797	E21.89950	116	200	5
2	Létavértes	N47.42269	E21.91110	117	200	4
3	Létavértes	N47.44133	E21.92817	120	130	2
4	Hajdúbagos	N47.41580	E21.68023	107	140	2
5	Hajdúbagos	N47.41183	E21.68336	109	140	2
6	Létavértes	N47.42478	E21.86181	116	200	5
7	Létavértes	N47.42928	E21.86382	114	200	5
8	Hajdúsámson (Martinka)	N47.57292	E21.77960	129	126	3
9	Hajdúsámson (Martinka)	N47.57391	E21.78137	130	126	3
10	Hajdúsámson (Martinka)	N47.57486	E21.79247	130	76	3
11	Monostorpályi	N47.40945	E21.77691	112	45	3
12	Monostorpályi	N47.41554	E21.78521	112	45	3
13	Hajdúsámson (Martinka)	N47.58068	E21.77093	132	57	4
14	Hajdúsámson (Martinka)	N47.57541	E21.79281	131	Grazing exclusion	1
15	Vámospércs	N47.53241	E21.95022	132	Grazing exclusion	1

### 2.2 Sampling

We sampled the biomass of 15 pastures from late May to early June 2021. The pastures were managed by seasonal sheep herding (Merino breed, typically from early April to the end of October). According to the national park rangers, the grazing intensity in any given site was similar between 2017 and 2021. Two sites had been fenced for 13 years (since the summer of 2008) to exclude livestock grazing (Aszalósné Balogh et al., 2023). The pastures were selected to cover a broad range of grazing intensity (Table 1) considering the stocking density, the proximity of (frequently used) drinking and/or resting places, the number of faeces (dropping events) and the physiognomy of the vegetation (for details see Tables 1 and 2). We also used satellite imaging to assess the distance to the resting/drinking places. We avoided sites where the prevailing disturbance was not grazing and sites with markedly different habitat types (e.g. forest plantations, dirt roads and drained low‐lying areas). Information on approximate grazing intensities provided by national park rangers were refined during the field sampling by interviewing the shepherds. National park rangers also helped in site selection and confirmed that the stocking density alone is not sufficient to assess the grazing intensity, an assumption also supported by some former studies (e.g. Tonn et al., 2019). Taking all this information into account, we classified our sampling sites into five grazing intensity categories (Table 2).

**TABLE 2:** Levels of grazing intensity depending on stocking density, proximity to resting and drinking places and units of faeces found per sampling site. One sheep is equivalent to 0.2 livestock units per hectare (LU/ha), and one unit of faeces is equal to one dropping event. Grazing intensity increases from ‘1’ to ‘5’, where ‘1’ means grazing exclosure and ‘5’ is the highest level of grazing intensity.

Level of grazing intensity	Stocking density (LU/ha)	Proximity to resting and drinking places (m)	Units of faeces (10 m × 10 m area)
1	Grazing exclusion		
2	0.5–0.8	>150	0–20
3	0.5–0.8	<150	>20
4	1.1–4	>150	0–20
5	1.1–4	<150	>20

In each pasture, we designated a 10 m × 10 m sampling site to ensure uniform biomass sample heterogeneity. Before the biomass sampling, we recorded the complete list of vascular plant species in each site to facilitate the biomass sorting in the laboratory. In each sampling site, we harvested the total aboveground biomass of ten 20 cm × 20 cm sampling plots (altogether 150 samples) using secateurs. Standing litter and the litter layer were also included in the samples. The samples were dried using a drying oven (65°C for 48 h). After drying the biomass was sorted into the main fractions moss, lichen, litter (including both the litter layer and standing litter) and green biomass. Green biomass was further sorted to vascular plant species level, whereas the moss and lichen fractions were not further differentiated. The sorted biomass fractions were weighed using an electronic balance (accuracy: ±0.01 g).

During the biomass sampling, we also collected soil samples from the upper 5 cm soil layer of each biomass sampling plot to characterise the average site properties. The ten samples per site were pooled to eventually obtain at least 500 g air‐dried soil for each sampling site. Soil samples were analysed by an accredited laboratory for physical soil type, pH, humus, NO_2_^−^ and NO_3_^−^, K_2_O, P_4_ O_10_, CaCO_3_ and total of water‐soluble salts (Appendix S1).

### 2.3 Data processing and analyses

For the functional analysis of the sorted biomass of vascular plant species, we obtained regional plant trait data from the Pannonian Database of Plant Traits (PADAPT, Sonkoly et al., 2023). We classified the species into simplified morpho‐functional groups of short‐lived forbs, short‐lived graminoids, perennial forbs and perennial graminoids according to PADAPT and Király (2009) and into social behaviour types (SBTs) following Borhidi (1995). SBT is a refined Grime's CSR classification scheme adapted to the Hungarian flora, which has a robust and expanded subclassification for the ruderal strategy type. Using the SBT classification system we grouped the species into three categories along a gradient of increasing disturbance tolerance: (1) sand grassland species including the categories competitors (C), specialists (S), generalists (G) and natural pioneers (NP) of sand grasslands, (2) natural disturbance‐tolerant species (DT) and (3) ruderal weedy species including the categories ruderal competitors (RC), 0000 (AC) and weeds (W). For each plot we calculated community‐weighted means (CWMs) of this ordinal variable (Groups 1, 2 and 3, respectively) weighted by biomass and used it as an ecological indicator of disturbance (disturbance index) in the analyses. We used generalised linear mixed‐effect models (GLMMs) to assess the impact of sheep grazing on dependent variables (intensity level included as fixed factor, site identity as random factor and dependent variables listed in Table 3; SPSS 26.0 program package, IBM Corp, 2019). We plotted species richness against green biomass and analysed their relationship using a second‐order polynomial fit. The biomass composition of sites and grazing intensities were explored by canonical correspondence analysis (CCA) using the CANOCO 5.0 programme package (Šmilauer & Lepš, 2014). We included seven variables (soil phosphorous content, pH, soil compactness, soil nitrogen content, soil potassium content, disturbance index and soil humus content) in the secondary explanatory matrix of the CCA and selected significant predictors by a Monte‐Carlo permutation test.

**TABLE 3:** Effects of the intensity of sheep grazing on species richness, disturbance and biomass fractions.

Variable	Grazing intensity	
	*F*_4,145_	*p*
Species richness	5.66	<.001
Disturbance	**6.24**	**<.001**
Main biomass fraction		
Green biomass	**2.49**	**.046**
Litter	**10.97**	**<.001**
Mosses + lichens	0.953	.435
Specific biomass fraction		
Perennial forbs	**25.88**	**<.001**
Perennial graminoids	**3.21**	**.015**
Short‐lived forbs	**11.20**	**<.001**
Short‐lived graminoids	0.27	.897

*Note:* Significant values (generalised linear mixed‐effect model (GLMM), *p* < .05) are highlighted in boldface.

## 3 RESULTS

**FIGURE 2:**
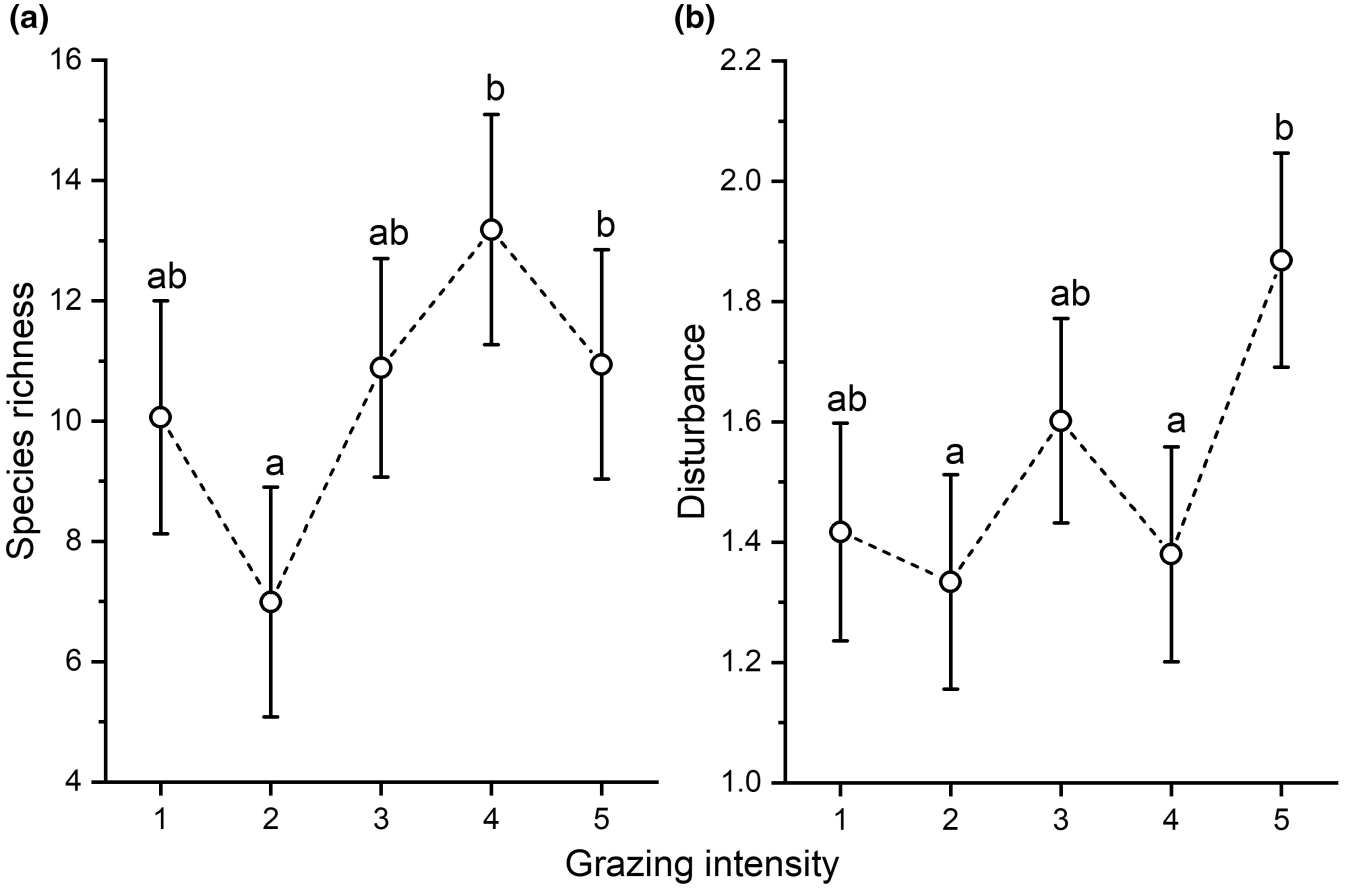
Species richness (a) and disturbance index (b) along a grazing intensity gradient obtained by a generalised linear mixed‐effect model (GLMM). Circles denote estimated means, whiskers standard errors. Different letters denote significant differences. The grazing intensity levels 1 to 5 are explained in Table 2.

**FIGURE 3:**
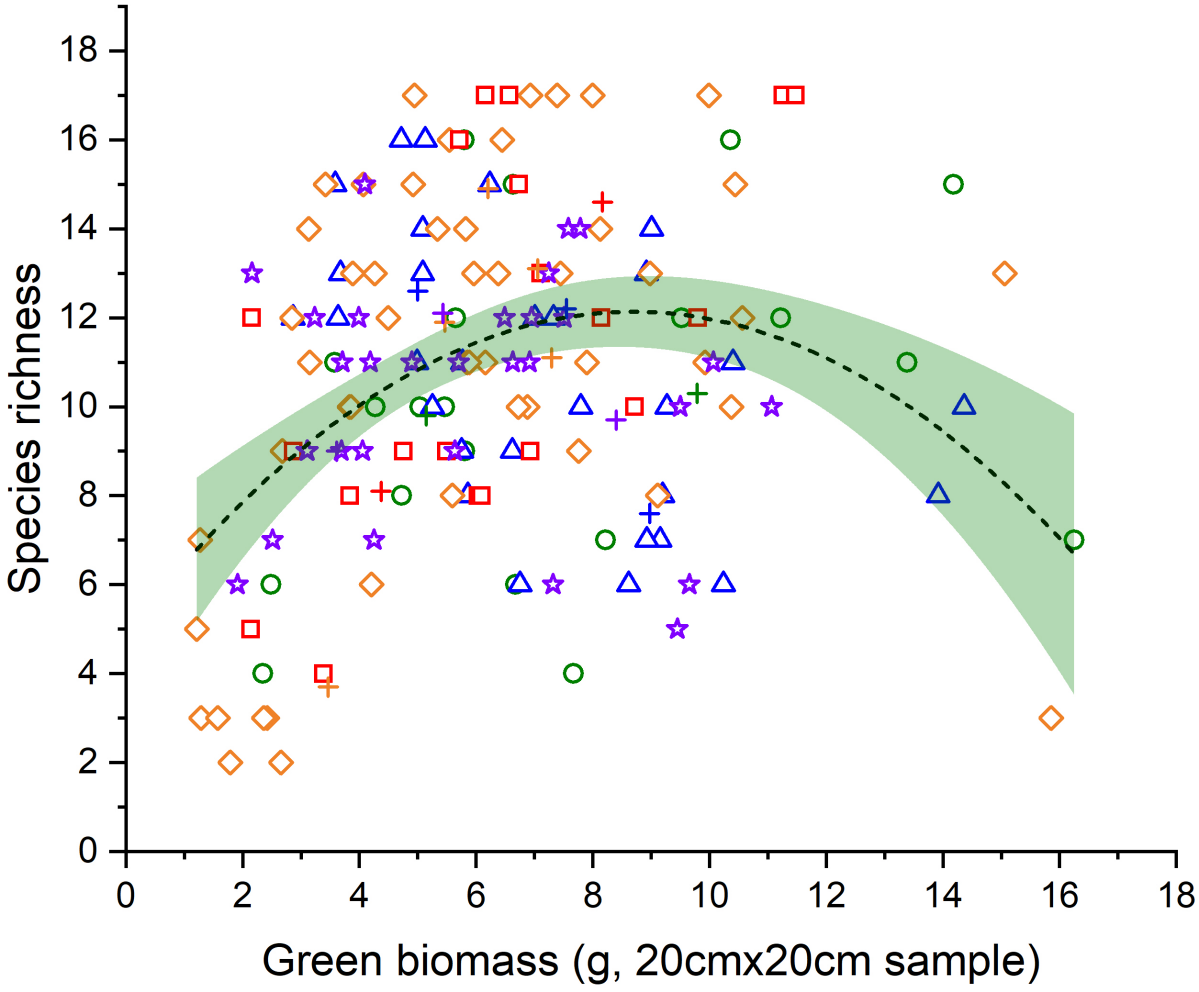
Relationship between the green biomass and species richness obtained by a second‐order polynomial fit (*p* < .001, *R* ^2^ = 0.143). The ‘+’ signs denote the centroids of the samples originating from the same site and grazing intensity, and all other symbols denote the estimated means of individual samples collected on site. The green shade indicates the 95% confidence interval. Different symbols and their colours denote the level of grazing intensity as follows: 1: Green circles, 2: Blue triangles, 3: Orange diamonds, 4: Red rectangles and 5: Violet stars. The levels of grazing intensity are explained in Table 2.

We detected 84 species in the samples consisting of 24 graminoids (8 short‐lived and 16 perennial) and 60 forbs (36 short‐lived and 24 perennial). Grazing intensity had a significant effect on green biomass, litter, the biomass of perennial forbs, perennial graminoids and short‐lived forbs and on species richness and disturbance index (Table 3). The species richness at the fourth and fifth level of grazing intensity was significantly higher than that at the second level, and we detected the highest species richness at the fourth level of grazing intensity (Figure 2a). The disturbance index was significantly higher at the fifth level compared to all other levels (Figure 2b). We detected a humped‐back relationship between the amount of green biomass and species richness (Figure 3).

**FIGURE 4:**
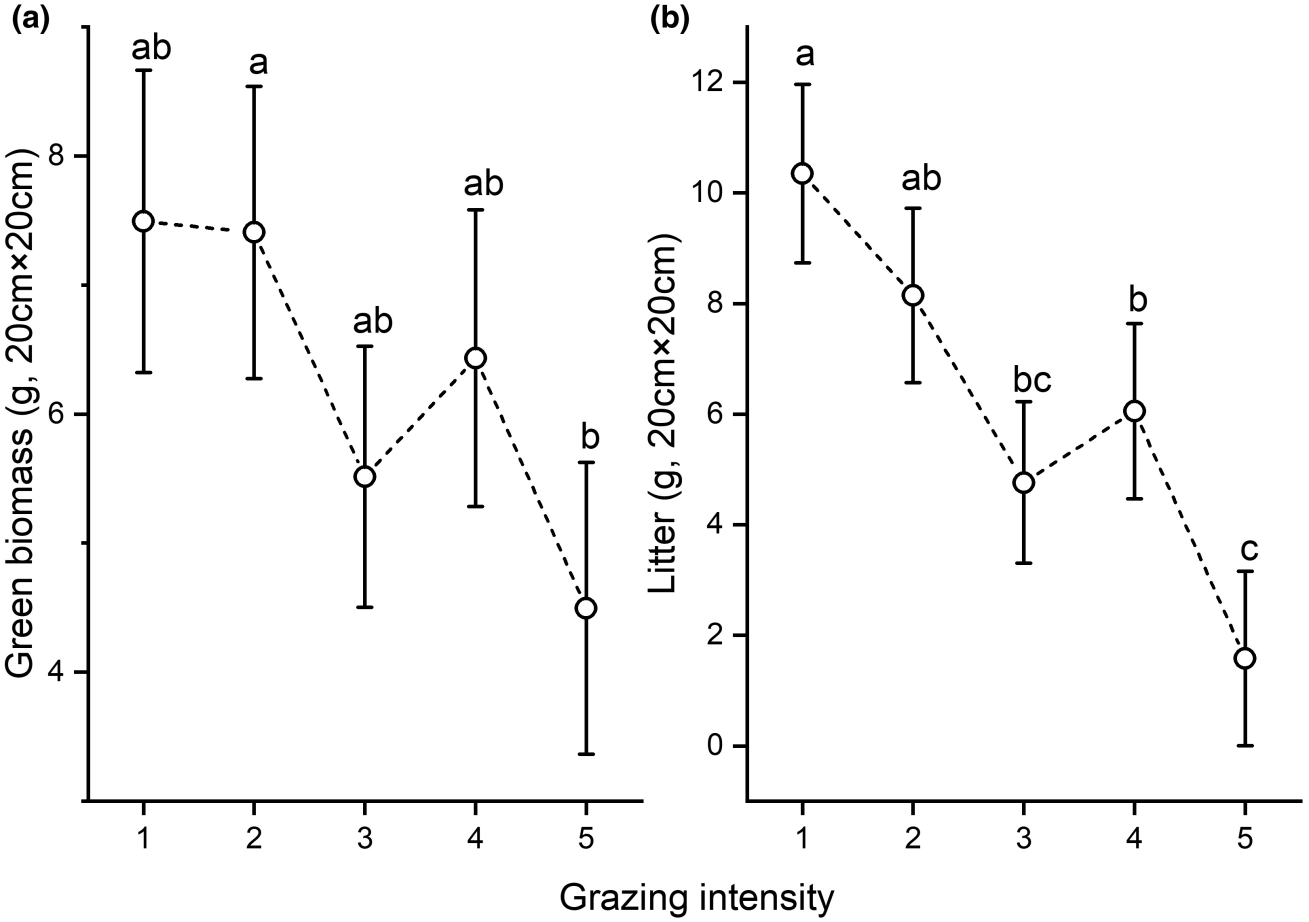
Green biomass (a) and litter (b) along a grazing intensity gradient obtained by a generalised linear mixed‐effect model (GLMM). Circles denote estimated means, whiskers standard errors. Different letters denote significant differences. The grazing intensity levels 1 to 5 are explained in Table 2.

**FIGURE 5:**
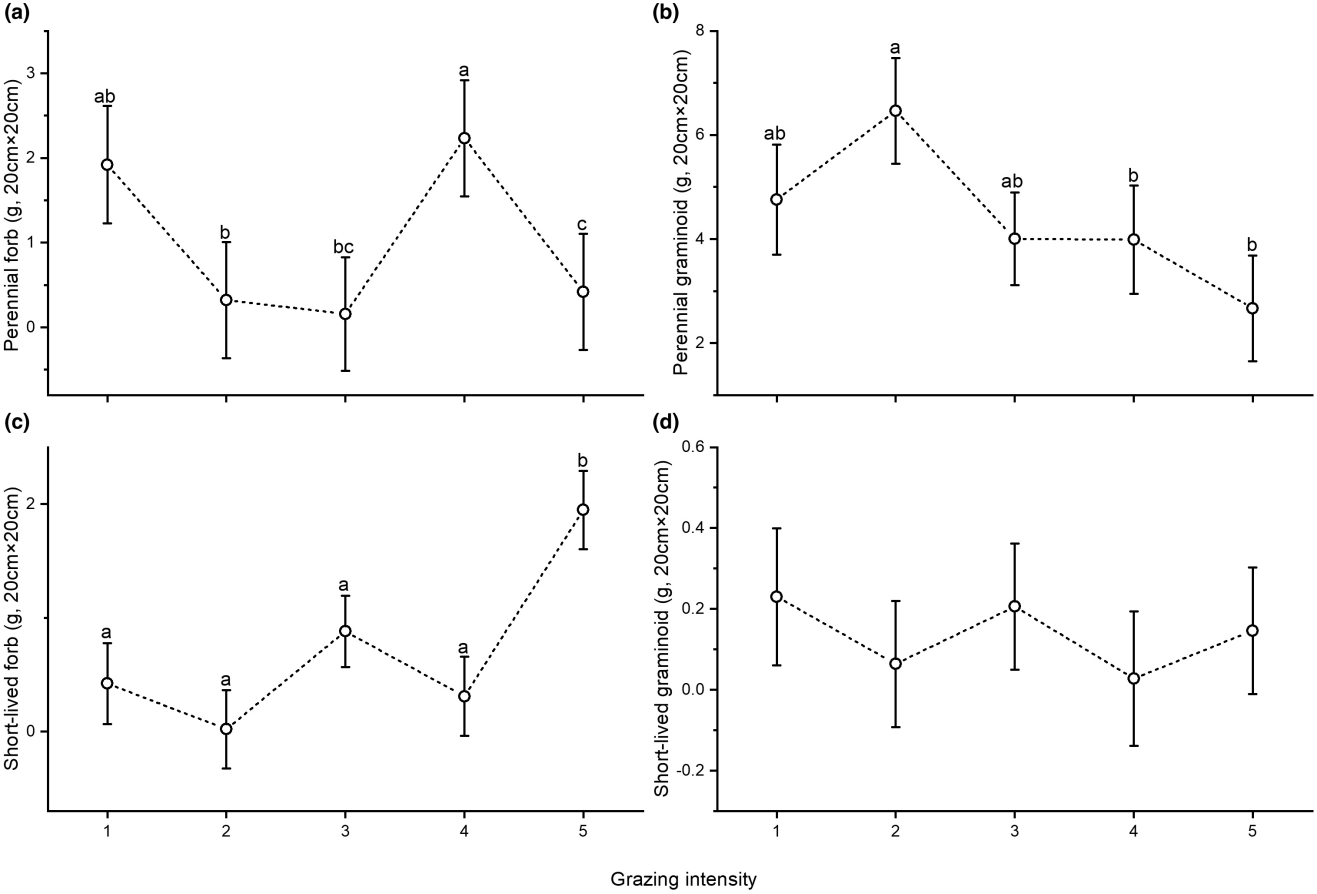
Biomass of perennial forbs (a), perennial graminoids (b), short‐lived forbs (c) and short‐lived graminoids (d) along a grazing intensity gradient obtained by a generalised linear mixed‐effect model (GLMM). Circles denote estimated means, whiskers standard errors. Different letters denote significant differences. The grazing intensity levels 1 to 5 are explained in Table 2.

We also detected significant differences for green biomass and litter. Both were highest at the first and lowest at the fifth level of grazing intensity (Figure 4a,b). Moss and lichen biomass did not show significant differences along the grazing intensity gradient. Perennial forb biomass was highest at the fourth level of grazing intensity (Figure 5a), whereas perennial graminoid biomass was significantly higher at the second than at the fourth and fifth level (Figure 5b). We found no significant differences in the biomass of short‐lived forbs between the first and fourth level of grazing intensity, whereas that at the fifth level was significantly higher compared to the other levels (Figure 5c). The biomass of short‐lived graminoids did not show significant differences (Table 3; Figure 5d).

**FIGURE 6:**
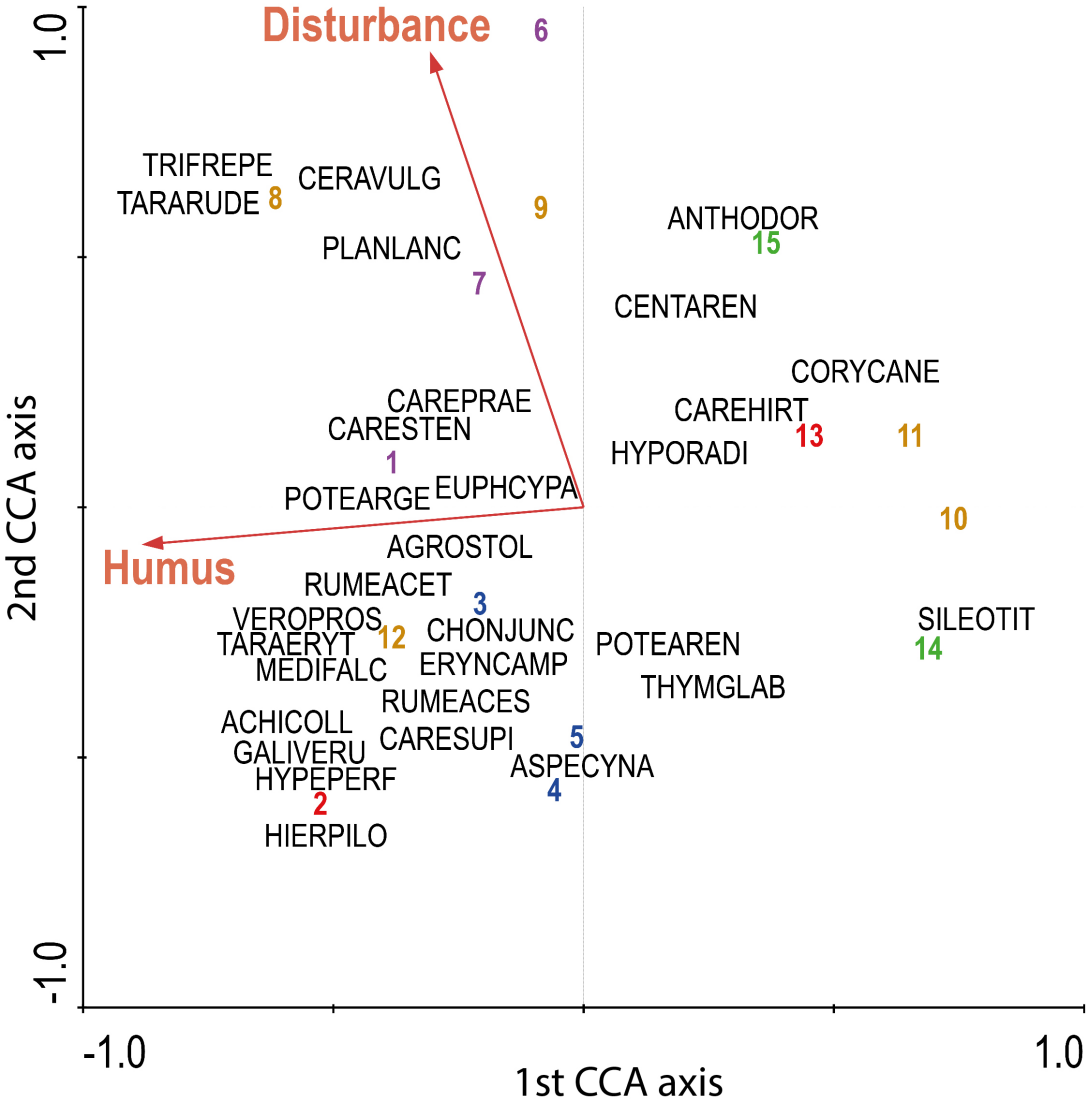
Relationship between species composition, humus content and level of disturbance (community‐weighted means (CWM) of social behaviour types (SBT), see text). For the canonical correspondence analysis (CCA), biomass per species was used. Eigenvalues were 0.617 and 0.504 for the first and second axes, respectively. The cumulative percentage variance of the species‐environment relation was 82.2 for the first four axes. Numbers denote the sampling sites (see Table 1) and their colours the levels of grazing intensity (1: Green, 2: Blue, 3: Orange, 4: Red and 5: Violet). Species names are abbreviated to the first four letters of the genus and the first four letters of the specific epithet. Full names of species are given in Appendix S3. Only significant explanatory variables are shown in the figure (499 permutations, *p* < .008).

The CCA identified only two significant predictors for the species compositional patterns, soil humus content and disturbance index, but these two predictors were weakly correlated with each other (Figure 6). We found no clear separation of pastures grazed at different levels of intensity based on the biomass composition, and sites with increasing levels of grazing intensity were also not separated along the predictors. Similarly, the samples of the different sites did not show any clear separation along either the axis of green biomass quantity or the axis of species richness (Figure 3).

## 4 DISCUSSION

### 4.1 Species richness and green biomass

We found a humped‐back relationship between species richness and green biomass. Moderate levels of disturbance provide favourable conditions for a wider range of species (Metera et al., 2010). The highest species richness was detected at the fourth and fifth grazing intensity levels. In accordance with our results several studies detected that species richness is higher under moderate grazing pressure than, for example, in ungrazed pastures (Deng et al., 2013; Fensham et al., 2011; Sasaki et al., 2009). Deng et al. (2013) found the density, the height and the cover of vegetation to be highest in the ungrazed plot, where also the densities of dominant, good competitor species were highest. When grazing intensity is low, more competitive plants can grow taller as they acquire more resources and can thus overcome resource limitation more effectively and grow efficiently in dense, ungrazed vegetation (He et al., 2021; Westoby, 1999). Livestock grazing decreases green biomass and litter (Magnano et al., 2019), and this effect is obviously more pronounced under more intensive grazing. This is also reflected in the high proportion of biomass samples belonging to the first and second level of grazing intensity at higher biomass values (Figure 3). Although we detected a humped‐back relationship between species richness and green biomass in the studied pastures, the possibility of a decreasing trend should not be excluded in unusually arid years (Gao & Carmel, 2020; Milchunas et al., 1988).

One would expect that the samples originating from the same grazing intensity levels would cluster in the ordination, but this was not validated by our results (Figure 6). The reason for this might be a fine‐scale heterogeneity of vegetation. Studying the vegetation composition of alkaline and sand pastures at multiple‐scales, Godó et al. (2017) observed that there is a significant relationship between plot size and grazing effects: With increasing plot size they found decreasing levels of differences in species composition, ie the small‐sized plots showed higher beta diversity than larger ones. We assume this to be the reason why our fine‐scale samples (harvested in 20 cm × 20 cm plots) were not clearly separated along the gradient of green biomass and species richness.

### 4.2 Main biomass fractions and the biomass of life forms

We found significant differences along the grazing intensity gradient for the main biomass fractions and the biomass of functional groups. Both the green biomass and the litter fraction were significantly lower at higher grazing intensities. Obviously, the consumption of plants by livestock contributes to a decrease in green biomass and litter (Magnano et al., 2019). According to Kemp et al. (2000) perennial graminoids are most sensitive to grazing, and with increasing grazing disturbance subordinated species are able to spread (Grime & Mackey, 2002). This might explain why we detected significantly lower amounts of perennial graminoids at higher grazing intensities. Green biomass showed significant differences, and though the biomass of short‐lived forbs was significantly higher at the highest grazing intensity, total green biomass decreased with increasing grazing intensity (as one would expect). There are several possible explanations for this pattern. First, sheep grazing may be less selective for forbs at higher grazing intensities (Golodets et al., 2009; Tóth et al., 2018), and the feeding strategy of sheep is flexible depending on the available biomass and/or forb richness of the pasture, displaying a mass foraging strategy in communities characterised by graminoids (Liu et al., 2015; Mládek et al., 2013). This causes a net loss of total green biomass, but at the same time favours short‐lived forbs due to their fast regrowth rate and colonisation ability in gaps (Hofmann & Isselstein, 2004; Westoby, 1999). Second, the mean height of species is typically lower at higher grazing intensities (Deng et al., 2013; Török et al., 2016) so that they may be represented by less biomass. Third, annuals have a higher specific leaf area (SLA) and a lower leaf dry matter content (LDMC) and thus a lower dry weight than other plants (with a lower SLA and a higher LDMC) (E‐Vojtkó et al., 2020). In their meta‐analysis of plant responses to grazing, Díaz et al. (2007) found that the abundance of perennial plant species decreased with increasing grazing intensity. This was also confirmed by our results to some extent, but a striking leap can be observed at the fourth level of grazing intensity for perennial forbs (Figure 5a). Among the detected perennial forbs *Thymus glabrescens* had remarkably high values at this grazing intensity level. Without this species there would be a continuous decline of perennial forb biomass along the gradient of grazing intensity. Their stout woody stems increase the biomass of perennial forbs, and their large amount of biomass shapes the differences of the total green biomass scores. *Thymus glabrescens* produces monoterpenes, which are known to act as feeding deterrents against sheep (Linhart & Thompson, 1999). It is therefore probably one of the less favoured food plants on the pastures we investigated. The meta‐analysis mentioned above (Díaz et al., 2007) details the response of annuals to grazing. It found them to increase together with grazing intensity, which is confirmed by our results in case of forbs. Another explanation for the spreading success of short‐lived plants is their generally higher SLA compared to perennials. A high SLA is linked to fast re‐growth ability (Helm et al., 2019), and higher SLA scores at higher grazing intensities were confirmed by a former study addressing livestock grazing in sand grasslands (Kovacsics‐Vári et al., 2023). We found no significant differences for short‐lived graminoids, which is presumably due to their low species number and the even distribution of the species along the gradient of grazing intensity. For example, *Apera spica‐venti* had higher biomass at lower levels of grazing intensity, whereas *Bromus hordeaceus* was stronger at higher grazing intensities.

### 4.3 Impact of grazing disturbance on species composition

Significantly higher biomass scores of disturbance‐tolerant and ruderal species were detected at the highest level of grazing intensity, where their species numbers were high, but they suppressed the biomass of characteristic species of sand grasslands. The process leading to overgrazing has been succinctly summarised by Schulze (2019): First, the vegetation composition changes; second, vegetation cover decreases; third, bare soil surfaces are formed and fourth, soil erosion becomes more severe. Our results might partly support this description, but we presume that these stages are not reached one after another, but occur in parallel. For example, when bare soil surfaces are formed due to the suppression of characteristic perennial grass and forb species, disturbance‐tolerant and ruderal species will take the opportunity and establish quickly, which changes the vegetation composition. Midolo et al. (2023) assessed plant species by disturbance categories and found that annuals are favoured by disturbance as their ability to grow fits to circumstances that do not provide stable biotic and abiotic features, which would otherwise favour better competitors using resources efficiently on the long‐run (Salguero‐Gómez, 2017; Schulze, 2019). According to Botta‐Dukát (2008), open sand grasslands are among the habitats most exposed to disturbance in Hungary. The Nyírség region has been densely populated for centuries, and despite the low productivity of sand grasslands, large areas were cultivated, resulting in high covers of disturbance‐tolerant and ruderal species. Pastures with open surfaces can quickly become colonised by these species, which typically have a good dispersal ability (Schulze, 2019), and according to our findings they even increased in species richness. This draws the attention to landscapes where pastures have to be sustainably managed in a densely populated region.

Our results showed that open surfaces and colonisation by disturbance‐tolerant and ruderal species are to be expected in intensively grazed pastures. The fourth and fifth level of grazing intensity included sampling sites with relatively high stocking densities (1.1 to 4 LU/ha), and the highest scores of species richness were observed in these pastures due to the higher number of short‐lived – mainly disturbance‐tolerant and ruderal – species. Increasing species richness in more intensively grazed sites was also confirmed by Kiss et al. (2006). In their assessment of grazing intensity,0 they considered the proximity of study sites to stables, and similar to us they detected a high proportion of disturbance‐tolerant and ruderal species contributing to the higher species richness. We detected significantly higher species richness at the fifth and fourth level of intensity compared to the second level. At the fourth level, the presence of grassland species was still substantial, but disturbance‐tolerant and ruderal species already occurred more frequently. One can find significant differences in most of the studied characteristics if the second and fourth levels of grazing intensity are compared to the third and fifth (Figures 1 and 3), which implies that the sites representing the third and fifth level were grazed and trampled more frequently. These significant differences suggest that besides the stocking density the proximity to resting and drinking places has a noticeable effect on the vegetation as well. We assume that a closer proximity to resting and watering points results in a higher grazing frequency and intensity. In their study on the effect of proximity to resting and drinking places on plant characteristics, Kovacsics‐Vári et al. (2023) found similar results for flowering period and life forms. In this context, it should be noted that the findings by Kiss et al. (2006) and Tonn et al. (2019) suggest that the effects of livestock grazing should be studied on finer scales than the scale of a pasture since the stocking density, as an important metric in grazing regimes, is an average figure for the entire pasture area and does not take local variations into account.

## 5 CONCLUSIONS

We found that disturbance‐tolerant and ruderal species contributed to the increase of species richness at higher levels of grazing intensity, but also suppressed some of the characteristic species, which might lead to decreasing stability and ecosystem functions. To interpret this finding data on proximity to frequently used places seemed useful. We hope that our results will help in the arrangement of pastures and be a decision‐making aid with regard to a site‐adapted stocking density in situations where conservation grazing is necessary to maintain characteristic and reduce the encroachment of non‐desired species. One implication of our findings is that resting and drinking places should not be placed close to the field margins and/or to roads or ruderal habitats, which could act as sources of weed propagules. We detected a humped‐back relationship between green biomass and species richness in the studied sand pastures, but the plots characterised by different grazing intensities were not clearly separated from each other along the biomass gradient. This clearly indicates that (i) levels of grazing intensity based on the stocking density on pasture scale are too robust to assess effects of grazing intensity on the vegetation and (ii) even sheep grazing created local variations in the vegetation with respect to species richness and biomass at the scale of the biomass sampling. These results suggest that we might need to use multiple scales for sampling and a fine‐scale assessment of grazing intensity. We formulated assumptions regarding the impact of proximity on grazing frequency, but to find clearer effects, a broader knowledge about each pasture would be needed.

## AUTHOR CONTRIBUTIONS

**Gergely Kovacsics‐Vári:** Conceptualization (supporting); data curation (equal); investigation (equal); methodology (supporting); project administration (supporting); validation (supporting); visualization (supporting); writing – original draft (supporting); writing – review and editing (supporting). **Judit Sonkoly:** Conceptualization (supporting); data curation (supporting); funding acquisition (supporting); investigation (supporting); validation (supporting); writing – review and editing (supporting). **Katalin Tóth:** Validation (lead); writing – review and editing (supporting). **Andrea McIntosh‐Buday:** Data curation (supporting); investigation (supporting); validation (supporting); writing – review and editing (supporting). **Patricia Elizabeth Díaz Cando:** Investigation (supporting); writing – review and editing (supporting). **Viktória Törő‐Szijgyártó:** Data curation (supporting); investigation (supporting); writing – review and editing (supporting). **Nóra Balogh:** Investigation (supporting); writing – review and editing (supporting). **Luis Roberto Guallichico Suntaxi:** Investigation (supporting); writing – review and editing (supporting). **Francis David Espinoza Ami:** Investigation (supporting); writing – review and editing (supporting). **Gábor Matus:** Resources (supporting); writing – review and editing (supporting). **Béla Tóthmérész:** Conceptualization (supporting); writing – review and editing (supporting). **Péter Török:** Conceptualization (lead); data curation (equal); formal analysis (equal); funding acquisition (lead); investigation (equal); methodology (lead); project administration (lead); validation (supporting); visualization (lead); writing – original draft (lead); writing – review and editing (equal).

## CONFLICT OF INTEREST STATEMENT

None declared.

## Supporting information


**Appendix S1.** Soil properties of the studied sand pastures.

**Appendix S2.** Raw biomass data of the grazed pastures.

**Appendix S3.** Species composition data of the grazed pastures.

## Data Availability

Underlying data are provided in Appendices [Link], [Link].
