# Supplementary material for: High species richness of sheep‐grazed sand pastures is driven by disturbance‐tolerant and weedy short‐lived species
Source: Ecol Evol. 2024 Sep 8;14(9):e70282. doi: 10.1002/ece3.70282 (PMC11381081; doi:10.1002/ece3.70282)
Supplement: Supplementary file 1 — Appendix S1. Soil properties of the studied sand pastures. [file ECE3-14-e70282-s002.doc]

**Appendix S1.** Soil properties of the studied sand pastures. The site codes are used in the canonical correspondence analysis (CCA) (Figure 5). Total water-soluble salt content and CaCO_3_ content were low and the same across all sample sites (total salt: <0.02 m/m%; CaCO_3_ <0.1 m/m %) and are therefore not shown in the table. Ranges of physical soil type (K_A_; physical soil texture analysis by Arany, scores are cm^3^ water uptake of 100g air-dried soil reaching a specific consistency level): 25> (coarse sand); 25–30 (sand); 31–37 (sandy loam); 38–42 (loam); 43–50 (clay loam); 51–60 (clay); 60< (heavy clay). K_2_O and P_4_O_10_ contents are measured in C_3_H_9_O_3_N-soluble; NO_2_^˗^ and NO_3_^˗^ in KCl-soluble form, respectively.

| **Site code** | **pH (KCl 1:2.5)** | | **Physical soil type [KA]** | **Humus**  **[m/m%]** | **NO_2_^˗^ and NO_3_^˗^**  **[mg/kg]** | **K_2_O [mg/kg]** | **P_4_O_10_**  **[mg/kg]** |
| --- | --- | --- | --- | --- | --- | --- | --- |
| 1 | 5.39 | 25 | | 1.5 | 2 | 85 | 40 |
| 2 | 4.74 | 25 | | 1.8 | 2 | 87 | 58 |
| 3 | 4.45 | 25 | | 1.6 | 3 | 65 | 59 |
| 4 | 5.18 | 27 | | 1.4 | 1 | 107 | 42 |
| 5 | 5.24 | 25 | | 1.3 | 1 | 105 | 34 |
| 6 | 5.24 | 25 | | 1.2 | 1 | 101 | 61 |
| 7 | 5.39 | 25 | | 1.7 | 2 | 121 | 85 |
| 8 | 5.71 | 28 | | 2.1 | 2 | 191 | 235 |
| 9 | 5.61 | 25 | | 1.4 | 2 | 111 | 41 |
| 10 | 5.49 | 25 | | 0.8 | 1 | 83 | 82 |
| 11 | 5.03 | 25 | | 0.9 | 3 | 53 | 42 |
| 12 | 7.26 | 32 | | 2.6 | 3 | 184 | 137 |
| 13 | 4.66 | 25 | | 0.7 | <1 | 58 | 39 |
| 14 | 5.01 | 25 | | 0.6 | 1 | 58 | 38 |
| 15 | 5.22 | 25 | | 0.8 | 1 | 75 | 40 |
